# Notes and News

**Published:** 1888-10-20

**Authors:** 


### Notes and News.

Collected and Communicated.

Both Bedford and Salford have secured sites for infectious hospitals, and will shortly commence building operations.

The total number of new students entered at University College Hospital for the current session is 153. Of these 65 are preparing for the preliminary scientific examination, 70 are registered as full students, and 18 are taking a portion of their curriculum. The progress of this medical school has been and continues to be very remarkable.

The number of female medical students entered at Paris this term is 114, of whom 90 are Russian, 12 French, and eight English, with one American, one Austrian, one Greek, and one Turk.

The new fever hospital at Walker is now closed to visitors and open to patients. A telephone has been placed between the wards and the porter's lodge, so that friends can talk to the patients without risk of infection.

It has been decided to erect a new public hospital at Dover, the cost of which will be upwards of £5,000. The new building will be adjacent to the old hospital, which was opened as a memorial at the general thanksgiving of 1854.

Mrs. Perry-Herrick has given £300 towards the building fund of the Leicester Children's Hospital. Mr. Cook, of excursionist fame, has promised an annual subscription of twenty-five guineas.

The Gresham Lectures on Physic this autumn dealt largely with the subject of "Voyages in Vacation," on which a series of articles are now appearing in our pages. As usual, Dr. Symes Thompson spoke well and clearly, and at the last lecture kindly answered all questions put to him.

"Ida," writing to the *Newcastle Weekly Chronicle*, says: "Will not any of your readers kindly inform me what they think of hospital nursing as a profession for ladies? As the letters on lady doctors have closed, I am sure a discussion on hospital nursing would interest many readers."

On October 9th the Earl of Verulam opened a new hospital which has been erected at St. Albans as a Jubilee memorial. The cost of the building is over £4,000, and twelve beds are ready for immediate use. The Earl of Verulam, Viscount Grimston, M.P., and Lord Grimthorpe afterwards spoke at a public luncheon at the County Club.

The Charing Cross Hospital Scholarship of fifty guineas, open to students of the Universities of Oxford and Cambridge, has been awarded to Mr. Albert Carling, of St. John's College, Cambridge. The Entrance Scholarship of one hundred guineas has been awarded to Mr. William Escombe, and that of fifty guineas to Mr. Percy J. Probyn.

On October 9th the foundation-stone of a new children's hospital for Leicester was laid by the mayor of the borough. The building is being erected as a wing of the present infirmary, and nearly £9,000 has already been subscribed for the purpose. The ceremony was performed with Masonic honours in the presence of a large company. The hospital will accommodate fifty children.

The unemployed are, with the approach of cold weather, beginning to make themselves heard again. The Board of Trade returns, however, show that business, both in London and the country, is much more active than it has been for some time, whilst in not a few industries higher wages are being paid and employment is more abundant. It may be hoped that practical steps will be taken for dealing with poverty at the outset, and that we shall have no repetition of the distress of last year.

The interesting ceremony of laying the foundation-stone of a new hospital for Sunnyside Asylum was performed by Provost Scott last week. The weather was delightful, the gathering of managers and friends being very large. The building is to cost from £12,000 to £15,000, and is to accommodate 100 patients. It is intended that the sick and the weakly get the benefit of this new building so far as they need it. Their transference will prevent overcrowding in the main building at Montrose.

A citizen of Salisbury, speaking of the Hospital Sunday of that city, says that "he had never seen so much drunkenness, or anything like so much, on a Sunday in Salisbury before." It seems, indeed, to be the case that Hospital Sunday was observed with a great deal of riotous and disgraceful behaviour. The verdict at which, according to the *Salisbury Times*, a considerable proportion of the people have arrived is, that Hospital Sunday has been "weighed in the balance and found wanting."

The report of the Enfield Jubilee Committee has been published. The spontaneous effort on the part of the people of Enfield to show their loyalty to the Sovereign found expression in something more than mere sentiment. The handsome sum of £665 was subscribed to make the auspicious event memorable for beneficent deeds. The most important of these Jubilee acts in Enfield was the building of a new wing to the Cottage Hospital, which is now completed, and reflects great credit upon all concerned. The wing was opened on Saturday, the 13th, by Mr. H. C. B. Bowles.

A sum of £114 has been forwarded to the secretary of the Great Northern Central Hospital, by Mr. Walter Madin, proprietor of the Eaglet Hotel, Seven Sisters Road. The amount represents the net proceeds arising from the recent athletic sports and fête held at Tufnell Park grounds on behalf of the Hospital. The fête was promoted by the principal tradesmen and the police of the Y division of Holloway. The licensed victuallers of the district took an active part in the promotion and success of the gathering. Mr. Walter Madin was honorary treasurer.

Swindon made a great demonstration over the opening of its Victoria Hospital. The evening after the ceremony the town was illuminated, and a torchlight masquerade procession passed through the streets. The procession presented a most novel and grotesque spectacle, the extraordinary blending of costumes of various nations and callings being as bewildering to the eye as the babel of cries and discordant instruments carried by the processionists was to the ear. The hospital van was an especial object of interest in the crowd. The evening was marred by some heavy showers of rain, but apart from this fact the whole of the proceedings were carried out with eminent success.

The following vacancies are announced this week, the amount of salary in each case being given after the name of the institution :—

**Resident Medical Officers.**

Essex Lunatic Asylum. Junior Assistant Medical Officer.—£100.
Hospital for Consumption and Diseases of the Chest. House Physician.
Salop Infirmary, Shrewsbury. Assistant House Surgeon.—Board, etc.
Weston-super-Mare Hospital and Dispensary. House Surgeon.—£60.
Leeds Infirmary. Resident Obstetric Officer ; also one House Physician and two House Surgeons.—Board, etc.
North-West London Hospital, Kentish Town. Senior Resident Medical Officer.
Seamen's Hospital. House Physician.—£75, board, &c.

**Non-resident Medical Officers.**

Ennis District Lunatic Asylum. Visiting Physician.—£100.
Great Northern Central Hospital. Out-Patient Physician and Out-Patient Surgeon.
St. Pancras and Northern Dispensary. Physician and Surgeon-Accoucheur.
Seamen's Hospital Dispensary. Surgeon, £63.
Bristol Dispensary. Medical Officer.

A farewell presentation was made to Mr. H. Burford Norman, F.R.C.S., on October 11th, on the occasion of his retirement from the active work of his profession and his departure from Portsmouth. A dinner was given by the South-East Hants District Medical Society, which is a branch of the British Medical Association, and after the usual toasts a very handsome silver tea and coffee service were presented to Dr. Norman. The subscribers also asked Dr. Norman to accept, on behalf of his wife, a small drawing-room clock, which they hoped would remind the owner of the sympathy, esteem, respect and friendship that the medical men of Portsmouth would ever entertain for her husband.

Special harvest festival services were held in the chapel of the Seamen's Hospital, Greenwich, on Sunday, October 14th, and on Monday, 15th. The preacher on the Sunday morning was the Rev. J. Elliott, of Christ Church, Deptford, and in the evening the sermon was preached by the Rev. Brooke Lambert, B.C.L., vicar of Greenwich. A plentiful supply of fruits and flowers had been sent by local friends, and were very prettily arranged in the chapel by the matron (Miss Cooke), the sisters, and nurses. The occasion was rendered still more interesting by the unveiling of a beautiful circular stained-glass window, representing the failing faith of Peter on the sea, which was executed by Mr. E. M. Smith, being the kind and generous gift of Mr. Henry C. Burdett, in commemoration of his long association with the institution. The services were all well attended both by the staff and patients. The fruits were subsequently given to the patients in their several wards.

We published a week or two ago a note to the effect that a hospital nurse had said : "I think people would send us some of their children's left-off clothes if only they knew how much we wanted them ; and it does not matter if they are old. Anything that will fit a child comes in useful ; old slippers would be especially useful. Care should be taken that nothing is sent about which there may be any possibility of infection." Apropos of this quotation, we have received a note from Miss R. Garrett, honorary secretary to a society of ladies which supplies clothing to the poor. Miss Garrett informs us that if the nurses or any other person interested in the matter would communicate with her at 12, Highbury Crescent, N., the needs spoken of may be to some extent met by her society. The only condition the society imposes is this : That those who desire garments shall apply for them by letter or post-card to the secretary of the society. Miss Garrett adds : "It is curious how few hospitals seem able to comply with this simple rule."

At the Jubilee of the Perth Infirmary, the chairman spoke in warm praise of the nursing : "As for Miss Logan and the staff of nurses under her, it was needless to say much. Their work was more than satisfactory—it was simply perfection." These sentiments were received with applause by a large and representative audience."

The proposal to appoint visiting physicians to lunatic asylums meets with little favour from the medical profession. There are in England at the present time several asylums in which the system has been tried and given up. In Ireland, the practice of appointing visiting physicians to asylums is by no means infrequent, but so far as experience shows, the results of treatment in that country do not surpass those obtained in England or Scotland. It does not, however, seem undesirable that more effective supervision of asylums than that which is secured by the occasional visits of the Commissioners of Lunacy should be universal.

The governors of the Meath Hospital have resolved that in future the session at the Medical School shall commence on October 1st instead of November 1st, as, heretofore, This is a step in the right direction, which, it may be hoped, will be universally followed in Ireland. The session in London commences on October 1st, which cannot be said to be a day too early ; but the Scotch Medical Schools, on the other hand, make a point of opening their session on November 1st. It should be remembered, however, that in Scotland the summer session has now become almost as important a period of medical study as the winter session. There is, therefore, perhaps, less reason for change in Scotland than elsewhere.

Mr. Alderman Spark, of Leeds, is a favourable specimen of the energetic Northerner. During the last few years, largely owing to his sensible and decided action, the working classes of the Metropolis of Cloth have bestirred themselves in all kinds of ways on behalf of the maintenance of the medical charities of the town. Workshop weekly collections have been systematically organised, boxes have been placed in every suitable part of the town, popular open-air concerts in Roundhay Park and elsewhere have been held, and as a result thousands of pounds have been annually collected where before there were not thousands of pence. A still more satisfactory fruit of these movements has been the arousing of a generous enthusiasm among the working people which has spread to all the other members of the community. As we point out elsewhere, the magnificent sum of £30,000 has been raised within a month for the extension of the Leeds General Infirmary.

The quarterly meeting of the trustees and subscribers o the Bristol Royal Infirmary was held on the 23rd ult., when the Rev. Nicholas Pocock said that for the first time in his recollection they would probably have a little money in hand at the end of the year, instead of being, as they were last year, considerably in debt to the treasurer. That was owing to the very large legacies they had received this year, but they hoped the public would not be too sanguine in their estimate of those. The money would carry them over the present year and possibly over next year, but it must not be forgotten that their regular income was not equal to their expenditure. During the twenty years he had sat on the board the building had been enlarged and renovated, at an expenditure of about £20,000, but they had not increased the subscriptions which came in annually, and if it were not for the casual donations now and again, and very occasional legacies, they should not be able to get on at all without appealing to the public. The expenses had increased very much, and they could not get on without an increase of £2,000 per year in the income. The expenditure was about £12,000, and the regular income could not be more than £10,000. There was no business of public interest.

